# CT-Based assessment of sarcopenia and its association with biologic treatment outcomes in Chinese Children with Crohn's disease

**DOI:** 10.3389/fnut.2025.1660731

**Published:** 2025-10-20

**Authors:** Cheng Guo, Yan Kong, Guoli Wang, Junye Du, Chang Yu, Jie Wu

**Affiliations:** Department of Gastroenterology, Beijing Children's Hospital, Capital Medical University, National Center for Children's Health, Beijing, China

**Keywords:** Crohn's disease, infliximab, computed tomography enterography, body composition, sarcopenia, children

## Abstract

**Background:**

Sarcopenia affects treatment outcomes in patients with Crohn's disease (CD), yet research focusing on pediatric populations is limited. This study aimed to determine the prevalence of sarcopenia among Chinese children with CD and to evaluate its impact on biologic therapy by analyzing body composition parameters based-on computed tomography enterography (CTE).

**Methods:**

Pediatric CD patients who underwent CTE and received infliximab (IFX) treatment between 2022 and 2025 were enrolled. Clinical, laboratory, and radiological data were collected. CTE was utilized to assess body composition. The control group consisted of children without inflammatory bowel disease (non-IBD) who underwent abdominal CT scans.

**Results:**

A total of 68 children with CD (mean age 11.89 years) were included. The prevalence of sarcopenia was significantly higher in the CD group compared to the 136 controls (33.8% vs. 10.2%, *P* = 0.001). Body mass index (BMI) was identified as the only protective factor against sarcopenia (OR 0.734, 95% CI 0.578–0.932, *P* = 0.005). Among CD patients, those with loss of response (LOR) to IFX had a significantly higher incidence of sarcopenia than those in remission (50% vs. 23.8%, *P* = 0.027). After treatment with infliximab combined with total exclusive enteral nutrition (EEN) or partial enteral nutrition (PEN) in 44 children, follow-up CTE showed a significant reduction in sarcopenia prevalence (47.7% vs. 25%, *P* = 0.027).

**Conclusion:**

Sarcopenia is common in Chinese children with CD and adversely affects the efficacy of biological therapy. CTE is a valuable tool for assessing sarcopenia in this population. Early detection and intervention may improve clinical outcomes for children with CD.

## 1 Introduction

Crohn's disease (CD) is a chronic inflammatory disease of the gastrointestinal tract that can lead to progressive intestinal damage and disability (1). The incidence of CD among children in middle-income countries is increasing annually (2). Due to reduced food intake, intestinal malabsorption, chronic protein loss through the feces, and increased energy demands from hypermetabolism (3, 4), children with CD may suffer from growth disorders, malnutrition, and sarcopenia, even during remission (5). In 2014, the Asian Working Group for Sarcopenia defined sarcopenia as an age-related decline in skeletal muscle mass as well as muscle function (defined by muscle strength or physical performance) (6). Sarcopenia has been associated with older age, but it is also a known complication of various chronic diseases, such as cirrhosis, intestinal disorders, chronic kidney disease, neurodegenerative diseases, and malignant tumors (7–17). Sarcopenia has been observed in both children and adult with CD (18–28). Sarcopenia affects the prognosis of CD and is recognized as a risk factor for surgery, hospitalization, and postoperative complications in patients with CD (29).

The primary challenge in defining sarcopenia in children lies in lacking a standardized definition and diagnostic criteria (30, 31). Children's skeletal muscle mass can be assessed using various methods, including bioelectrical impedance analysis (BIA) (21), dual-energy X-ray absorptiometry (DXA) (17), computed tomography (CT) (17), and magnetic resonance imaging (MRI) (19). However each technique exhibits considerable variability and lacks established reference standards. The total skeletal muscle area measured by CT or MRI at the L3-L4 lumbar spine levels is regarded as the gold standard for assessing skeletal muscle mass (6). CT enterography (CTE) is a routine diagnostic procedure for children with CD, and can be utilized to evaluate their skeletal muscle mass without the need for additional tests. However, pediatric studies using CTE to assess muscle mass in CD patients are limited, and there are regional and racial variations in health standard reference values (31).

The number of relevant studies on sarcopenia in children with CD is limited, and there is a lack of longitudinal follow-up examining the interaction between sarcopenia and CD treatment. Therefore, this study aims to investigate the incidence of sarcopenia in Chinese children with CD using CT-based body composition parameters, observe the interaction between sarcopenia and CD treatment, and determine whether sarcopenia affects the response to biologic therapies.

## 2 Materials and methods

### 2.1 Study population

This study is a retrospective analysis conducted from January 2022 to February 2025, involving patients diagnosed with CD at the Gastroenterology Department of Beijing Children's Hospital. The inclusion criteria were as follows: (1) age under 18 years; (2) diagnosis of CD based on the revised Porto criteria (32); (3) completion of a CTE examination within 3 months prior to CD diagnosis; (4) received standard infliximab (IFX) treatment with a follow-up period exceeding 12 months; (5) availability of complete clinical data and clear CTE images suitable for body composition evaluation. For the control group, inclusion criteria included: (1) healthy children who underwent an abdominal CT scan at our center; (2) normal abdominal CT scan results; (3) sex and age were matched to the CD group; and (4) absence of chronic conditions such as cancer, chronic inflammatory diseases, infections, or malnutrition.

### 2.2 Data collection

We collected demographic and clinical data of CD patients from electronic medical records. The data included age, gender, body weight, height, body mass index (BMI), disease duration, extraintestinal manifestations (EIM), perianal lesions, Paris classification (33), Pediatric Crohn's Disease Activity Index (PCDAI) (34), disease activity, erythrocyte sedimentation rate (ESR), C-reactive protein (CRP), fecal calprotectin (FC) and response to IFX.

### 2.3 Definitions

BMI is calculated by dividing weight (kg) by the square of height (m). Based on the results of a blood routine examination, calculate the neutrophil-to-lymphocyte ratio (NLR), platelet-to-lymphocyte ratio (PLR), monocyte-to-lymphocyte ratio (MLR), neutrophil+monocyte/lymphocyte ratio (NMLR). The new systemic immune inflammation index (SII) was calculated using the formula platelet count × neutrophil count/lymphocyte count.

Primary non-response (PNR) refers to the lack of response to IFX treatment in children with CD by the 14th week of therapy; These patients do not achieve clinical remission or sufficient improvement and subsequently discontinue IFX. Secondary loss of response (SLOR) describes the discontinuation of IFX maintenance therapy following disease recurrence after an initial positive response, which may involve dose escalation (either increasing the dose or shortening the dosing interval). In this study, loss of response (LOR) encompasses both PNR and SLOR.

The induction dose of IFX was 5 mg/kg, administered intravenously at weeks 0, 2, and 6. During the maintenance period, IFX was given intravenously every 4 to 8 weeks.

The L_3_ Skeletal Muscle Index (L_3_SMI) is calculated by dividing the total cross-sectional area of all skeletal muscles at the L_3_ vertebral body level, including the psoas major, erector spinae, quadratus lumborum, transversus abdominis, external oblique, and internal oblique, measured via CT or MRI by the square of the individual's height in meters. The L_3_SMI Z-score was evaluated using previously published online tools (34) (https://square.umin.ac.jp/ped-muscle-calc/index.html, accessed on January 10, 2025). Sarcopenia was defined as an L_3_SMI Z-score less than −2 standard deviation, according to previous studies (16, 20).

### 2.4 Skeletal muscle area

**Figure 1:**
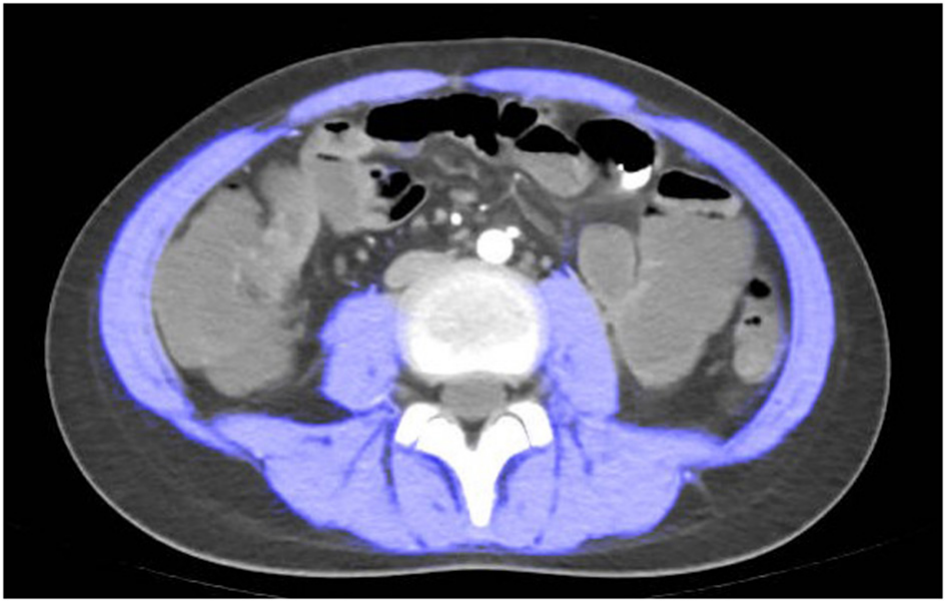
Computed tomography (CT) scan measured at spinal level L3. a patient with sarcopenia (Male, height = 1.55 m, L3SMI 28.67, L3SMI Z-score−2.423).

CTE examination was performed following the standard imaging protocol. At the level of the third lumbar vertebra, the boundaries of skeletal muscles were manually outlined using ImageJ software version 1.54p (35). Throughout the study, the boundaries of skeletal muscles were determined by trained radiologists. Pre-defined radiation attenuation ranges are used to demarcate muscle (-29 Hounsfield units to +150 Hounsfield units) (see Figure 1). Calculate the skeletal muscle area (SMA) in square centimeters from the pixel count using ImageJ.

### 2.5 Statistical analysis

Categorical variables are represented as frequency and percentage (%). Continuous variables are expressed as mean ± standard deviation or as median with interquartile range (IQR, lower and upper quartiles). Fisher's exact test was used to compare categorical variables, while *t*-tests or the Mann-Whitney U test were applied to compare continuous variables. Relevant indicators between clinical characteristics and sarcopenia/LOR were identified through univariate analysis, and followed by binary logistic regression analysis. A two-sided *P*-value < 0.05 was considered statistically significant. Statistical analyses were conducted using SPSS 26.0 (SPSS, Inc., Chicago, IL, USA). The Kaplan Meier curves generated with GraphPad Prism 10 (GraphPad Software, CA, USA).

### 2.6 Ethical approval

This study was approved by the Medical Ethics Committee of Beijing Children's Hospital in China (Approval Number: 2024-Y-073-D). Given the retrospective nature of the study, the requirement for informed consent was waived.

## 3 Results

### 3.1 Patient population

A total of 128 patients with CD completed CTE. Of these, 68 patients met the inclusion criteria and were included in this study. The mean age was 11.9 years (SD 9.5). Forty-two (61.8%) patients were boys. The median disease duration was 8 months (IQR, 2.3–11.3 months). Thirty-two patients (47.1%) had an ileo-colonic localization. 66.2% of patients received exclusive enteral nutrition (EEN), while 33.8% received partial enteral nutrition (PEN). Demographic data are presented in Table 1. Based on the L_3_SMI Z-score, 23 patients (33.8%) were diagnosed with sarcopenia. Two patients exhibited sarcopenia during the remission period. One in nine overweight or obese patients had sarcopenia, while six out of 32 children with normal nutritional status had sarcopenia.

**Table 1:** Demographic data.

**Variable**	**Value**
Age, years, median [M(SD), year]	11.9 (9.5)
Male, (*n*, %)	42 (61.8)
Disease duration [M(IQR), months]	8 (2.2–11.3)
BMI [M(IQR), kg/m^2^]	16.5 (12.4–18.0)
**Nutritional status, (** ***n*** **, %)**	
Malnutrition	27 (39.7)
Normal nutrition	32 (47.1)
Overweight or obese	9 (13.2)
ALB [M(IQR), g/dL]	3.7 (3.3–4.1)
*CRP* [M(IQR), mg/L]	7 (2–16.7)
Vitamin D [M(IQR), ng/mL]	36.5 (30.6–46.6)
*PCDAI* [M(IQR)]	32 (15.6–39.4)
**Disease location, (** ***n*** **, %)**	
L1 (terminal ileum)	6 (8.8)
L2 (colon)	7(10.3)
L3 (ileocolonic)	32 (47.1)
Upper GI involvement	23 (33.8)
Perianal disease, (*n*, %)	12 (17.6)
**Disease behavior, (** ***n*** **, %)**	
B1(non-stenosing nonpenetrating)	47 (69.1)
B2(stenosing)	14 (20.6)
B3(penetrating)	4 (5.9)
B2B3	3 (4.4)
**Disease activity, (** ***n*** **, %)**	
Remission	9 (13.5)
Mild	24 (35.3)
Moderate/severe	35 (51.2)
L_3_SMI[M(IQR), cm^2^/m^2^]	27.1 (23.8–31.9)
**Treatment, (** ***n*** **, %)**	
Corticosteroid	14 (20.5)
EEN	45 (66.2)
PEN	23 (33.8)
Sarcopenia, (*n*, %)	23 (33.8)

BMI, Body Mass Index; CRP, C-reactive protein; PCDAI, Pediatric Crohn's Disease Activity Index; L_3_SMI, skeletal muscle index at the third lumbar vertebra;EEN, exclusive enteral nutrition, PEN, partial enteral nutrition.

A total of 136 healthy children were included in the control group, all of whom underwent abdominal CT due to digestive foreign bodies, back pain or acute trauma. There were no significant differences in age, gender, BMI, or Vitamin D between the control group and the CD group (*P* > 0.05, Table 2). However, the L_3_SMI and L_3_SMI Z-score in the CD group were significantly lower than those in the control group (*P* < 0.001 and *P* = 0.002, respectively). Additionally, the incidence of sarcopenia was significantly higher in the CD group compared to the control group (33.8% vs. 10.2%, *P* = 0.001).

**Table 2:** Comparison between CD group and control group.

**Variable**	**CD**	**Control group**	***P*-value**
Age [M (SD), year]	11.89 (9.55)	11.14 (2.93)	0.104
Male*, n* (%)	42 (61.8)	77 (56.6)	0.54
BMI [M(IQR), kg/m^2^]	16.5 (12.4–18.0)	16.4 (14.7–19.7)	0.101
Vitamin D[M(IQR), ng/mL]	36.5 (30.6–46.6)	36 (33.7–39.5)	0.711
L_3_SMI [M(IQR), cm^2^/m^2^]	27.1 (23.8–31.9)	30.3 (27.1–33.3)	< 0.001
L3SMI Z-score [M(IQR)]	−1.65 (−2.31 to −1.24)	−0.98 (−1.58 to −0.42)	0.002
Sarcopenia, (*n*, %)	23 (33.8)	14 (10.2)	0.001

BMI, Body mass index; L_3_SMI, skeletal muscle index at the third lumbar vertebra.

### 3.2 Comparison between CD patients with sarcopenia and those without sarcopenia

**Figure 2:**
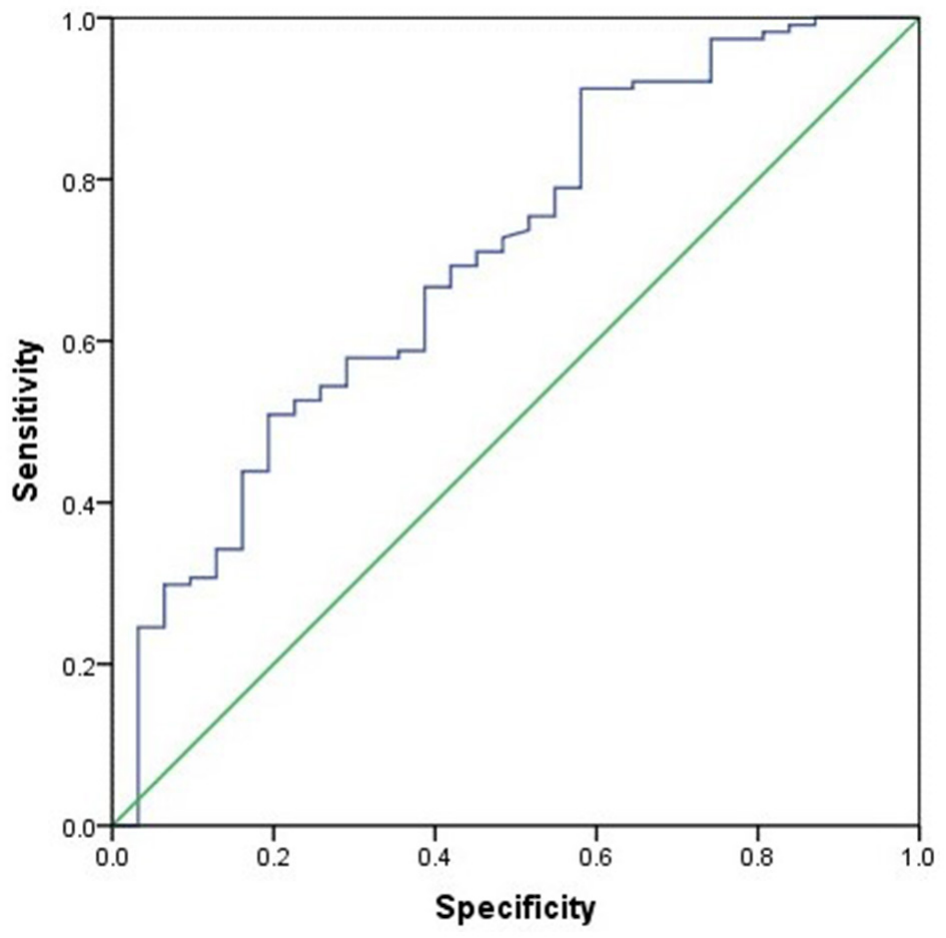
ROC curve of BMI prediction for sarcopenia.

The differences between CD patients with sarcopenia and those without sarcopenia are presented in Table 3. Compared to CD patients without sarcopenia, those with sarcopenia had significantly lower BMI, L_3_SMI, L_3_SMI Z-score, and proportions of normal nutrition and malnutrition (*P* ≤ 0.001). NLR, PLR, NMLR, and SII were significantly higher (*P* < 0.05), and the proportion of LOR to IFX treatment was also greater (*P* = 0.031) (Table 3). There was no statistically significant difference between the two groups in terms of corticosteroid use within 1 year prior to CD diagnosis and baseline vitamin D levels (measured before CD diagnosis). Subsequently, age, gender, BMI, NLR, PLR, NMLR, and SII were included in a binary logistic regression analysis, which identified that BMI (OR 0.734, 95% CI 0.578–0.932) as the sole protective factor against sarcopenia (*P* = 0.005). BMI showed the best performance in distinguishing the presence of sarcopenia, with an AUC of 0.731 (95% CI 0.60–0.85, *P* = 0.002). The optimal BMI cutoff value was 13.84, with a sensitivity of 79.1% and a specificity of 52.3% (see Figure 2).

**Table 3:** Comparison between CD patients with sarcopenia and CD patients without sarcopenia.

**Variable**	**CD patients without sarcopenia (*n* = 45)**	**CD patients with sarcopenia (*n* = 23)**	***p***
Age[M(IQR), year]	11.62 (10.7–13.7)	12.4 (11.6–13.0)	0.321
Male, n (%)	30 (66.7)	12 (52.17)	0.245
Disease duration[M(IQR), month]	6 (2–11)	6 (4–12)	0.366
Height[M(IQR), meter]	1.55 (1.34–1.63)	1.54 (1.4–1.6)	0.907
Weight[M(IQR), kg]	41 (27.25–48.5)	30 (25–42)	0.133
BMI [M(IQR), kg/m^2^]	16.4 (14.8–18.9)	14.3 (12.4–16.2)	0.002
L_3_SMI [M(IQR), cm^2^/m^2^]	30.8 (27.1–33)	20.8 (18.9–24.1)	< 0.001
L3SMI Z-score [M(IQR)]	−1.4 (−1.6 to −0.8)	−2.7 (−3.2 to −2.3)	< 0.001
Nutritional status, (*n*, %)			0.001
Malnutrition	11 (24.4)	16 (69.5)	
normal nutrition	26 (57.8)	6 (26.1)	
overweight or obese	8 (18.8)	1 (4.3)	
Extraintestinal manifestations, (*n*, %)	7 (15.5)	2 (8.6)	0.681
Anemia, (*n*, %)	29 (64.4)	19 (51.1)	0.12
CRP[M(IQR), mg/l]	7 (4–16.5)	7 (3–17)	0.82
HGB [M(IQR), g/l]	111 (102.5–126.5)	103 (96–115)	0.057
ALT[M(IQR), U/L]	9.7 (6.7–17.9)	10.6 (7–21.2)	0.509
ESR[M(IQR), mm/h]	20 (8.5–45)	18 (12–28)	0.825
ALB[M(IQR), g/l]	37.8 (34.85–40.4)	38 (34.2–41.2)	0.979
Vitamin D[M(IQR), ng/mL]	36.2 (30.8–46.4)	40.9 (30.3–47)	0.871
NLR[M(IQR)]	1.69 (1.09–2.76)	2.93 (1.97–4.04)	0.005
PLR[M(IQR)]	165.92 (114–247)	223 (174–296)	0.021
MLR[M(IQR)]	0.25 (0.17–0.36)	0.3 (0.21–0.44)	0.202
NMLR[M(IQR)]	1.43 (0.94–2.12)	2.38 (1.58–2.84)	0.003
SII[M(IQR)]	699.7 (367–1214.4)	1,396.3 (736.7–2,054.7)	0.005
Fecal calprotectin[M(IQR), mg/g]	1,786 (831–1,951)	1,800 (1,693–2,500)	0.057
Perianal disease, (*n*, %)	9 (20)	3 (13)	0.707
PCDAI[M(IQR)]	27.5 (15–35)	36.2 (20–57)	0.078
Disease location, (*n*, %)			0.082
L1 (terminal ileum)	4 (8.9)	2 (8.7)	
L2 (colon)	7 (15.6)	0 (0)	
L3 (ileocolonic)	21 (46.7)	11 (47.8)	
Upper GI involvement	13 (28.9)	10 (43.5)	
Disease behavior*, n* (%)			0.322
B1(non-stenosing nonpenetrating)	32 (71)	15 (65)	
B2(stenosing)	8 (18)	6 (26)	
B3(penetrating)	2 (4)	2 (9)	
B2B3	3 (7)	0 (0)	
Corticosteroid use within the past year	9 (20)	5 (21.5%)	0.867
PNR, (*n*, %)	6 (13.3)	7 (30.4)	0.09
LOR, (*n*, %)	13 (28.9)	13 (56.5)	0.031

IQR, interquartile range; BMI, Body Mass Index; CRP, C-reactive protein; PCDAI,Pediatric Crohn's Disease Activity Index; L3SMI, L3 skeletal muscle index at the third luminal vertebra; ALT, Alanine aminotransferase; ESR, erythrocyte sedimentation rate; ALB, Albumin; NLR, neutrophil to lymphocyte ratio; PLR, Platelet to lymphocyte ratio; MLR, Ratio of nuclear cells to beta cells; NMLR (neutrophil+monocyte)/lymphocyte ratio; SII, New systemic immune inflammatory index; L1, Distal 1/3 ileum ± limited ileocecal disease; L2, Colon; L3, Returning colon; B1, Non narrow and non penetrating diseases; B2, Narrowing disease; B3, Penetrating diseases; B2B3, Narrow and penetrating diseases; PNR, Primary loss of response; LOR, Lost response.

### 3.3 Predictors of Response to IFX Treatment

**Figure 3:**
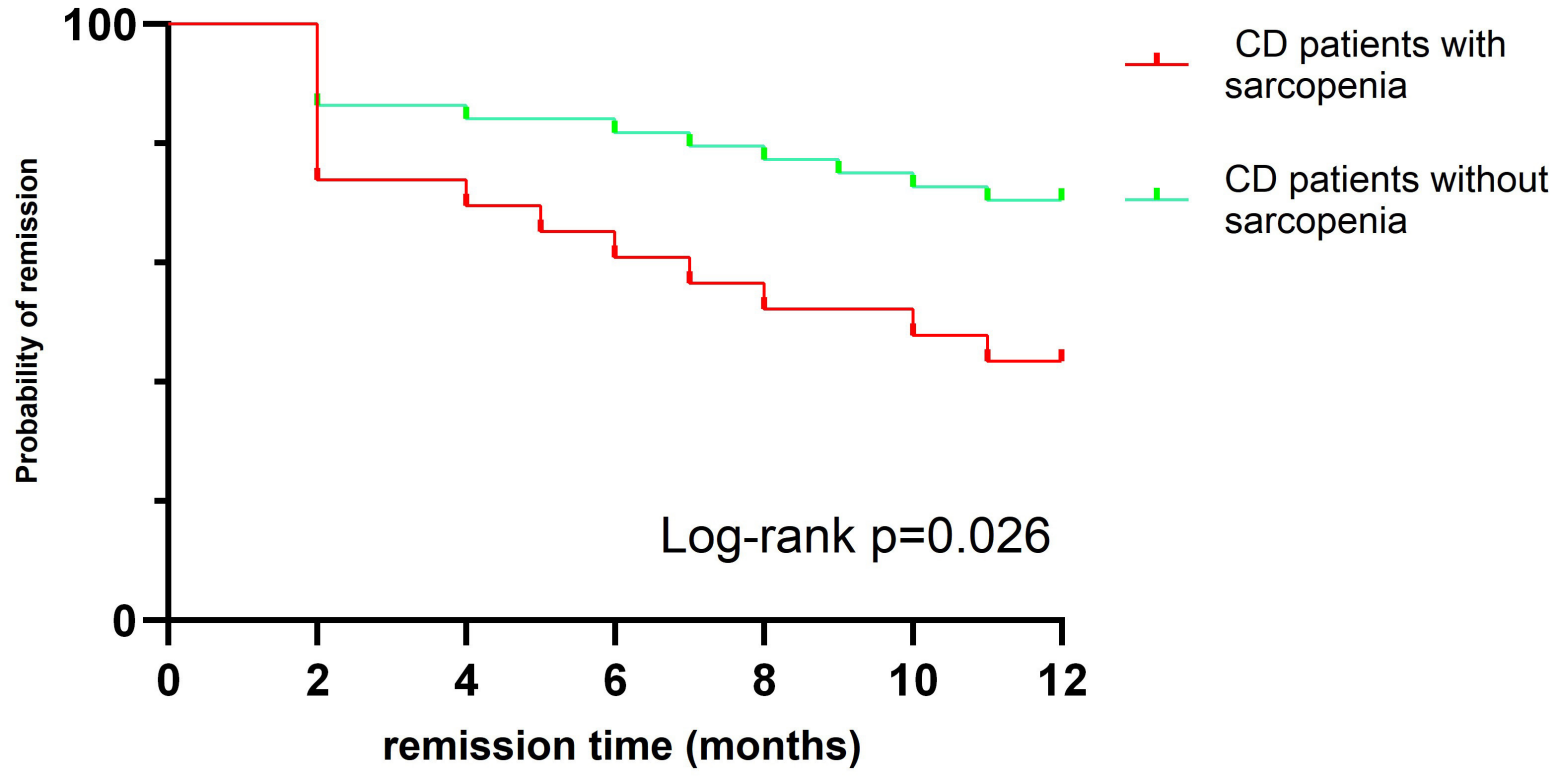
Kaplan–Meier curve showing different rates of remission between sarcopenia CD patients and No-sarcopenia CD patients.

After a one-year follow-up, 26 children who received IFX treatment were found to have LOR. The incidence of sarcopenia in LOR patients was significantly higher than that in remission group (50% vs. 23.8%, *P* = 0.027) (Figure 3). Baseline weight, BMI, ALT, and ALB levels were significantly lower in LOR patients than in those in remission (*P* < 0.05), while FC and PCDAI levels were higher in the LOR (*P* < 0.05) (see Table 4). Binary logistic regression analysis identified that PCDAI as an independent risk factor for loss of response to IFX treatment (OR=1.066, 95% CI 1.027–1.107, *P* = 0.001).

**Table 4:** Clinical characteristics related to IFX treatment response.

**Variable**	**Remission group (n = 42)**	**LOR group (n = 26)**	**P**
Age [M(IQR), year]	12.45 (11.3–13.7)	12.05 (10.52–13.52)	0.532
Male, (n, %)	25 (59.5%)	17 (65.4%)	0.629
Disease duration [M(IQR), months]	6 (2.75–10.5)	8 (2–12)	0.474
Height [M(IQR), meter]	1.55 (1.38–1.62)	1.45 (1.35–1.59)	0.185
Weight [M(IQR), kg]	41.3 (28.87–48.25)	28.15 (22.75–42.93)	0.024
BMI [M(IQR), kg/m^2^]	16.25 (14.85–18.73)	14.7 (12.43–16.55)	0.021
L_3_SMI [M(IQR), cm^2^/m^2^]	27..3 (25.5–32.3)	24.85 (19.97–31.9)	0.127
L3SMI Z-score [M(IQR)]	−1.59 (−1.96 to −1.24)	−2.11 (−2.78 to −1.05)	0.221
Sarcopenia, (n, %)	10 (23.8%)	13 (50%)	0.027
Nutritional status, (n, %)			0.153
Malnutrition	13 (31%)	14 (53.8%)	
Normal nutrition	22 (52.4%)	10 (38.5%)	
Overweight or obese	7 (16.7%)	2 (7.7%)	
Extraintestinal manifestations, (n, %)	5 (11.9%)	4 (15.4%)	0.965
Anemia, (n, %)	29 (69%)	19 (73.1%)	0.723
CRP[M(IQR), mg/l]	7 (3.75–13.25)	6 (3.75–30.25)	0.909
NLR[M(IQR)]	1.95 (1.46–2.78)	2.7 (1.06–5.54)	0.216
SII[M(IQR)]	744.4 (419.6–1343.5)	1127.2 (465.6–2363.3)	0.13
HGB [M(IQR), g/l]	111 (103.75–122.25)	103 (97.25–127.25)	0.218
ALT[M(IQR), U/L]	12.65 (9.02–20.45)	7.7 (5.87–12.95)	0.022
ESR[M(IQR), mm/h]	18 (11–39.5)	19.5 (7.75–34.25)	0.880
ALB[M(IQR), g/l]	38.4 (35.57–41.62)	36.1 (34.02–38.87)	0.044
Vitamin D[M(IQR), ng/mL]	36.3 (31.7–45.25)	38.45 (25–48.2)	0.663
Calprotectin[M(IQR), mg/g]	1776 (783–1800)	1830 (1679–1964)	0.037
Perianal disease, (n, %)	7 (16.7%)	5 (19.2%)	1
PCDAI, [M(IQR)]	23.75 (15–35)	35.25 (18.75–55.5)	0.001
Disease location, (n, %)			0.730
L1 (terminal ileum)	4 (9.5)	2 (7.7)	
L2 (colon)	3 (7.3)	4 (15.4)	
L3 (ileocolonic)	21 (50)	11 (42.3)	
Upper GI involvement	14 (33.3)	9 (34.9)	
Disease behavior, (n, %)			0.745
B1 (non-stenosing nonpenetrating)	30	17	
B2 (stenosing)	7	7	
B3 (penetrating)	3	1	
B2B3	2	1	

IQR, interquartile range; BMI, Body Mass Index; CRP, C-reactive protein; PCDAI,Pediatric Crohn's Disease Activity Index; L3SMI, L3 skeletal muscle index at the third luminal vertebra; ALT, Alanine aminotransferase; ESR, erythrocyte sedimentation rate; ALB, Albumin; NLR, neutrophil to lymphocyte ratio; PLR, Platelet to lymphocyte ratio; MLR, Ratio of nuclear cells to beta cells; NMLR (neutrophil+monocyte)/lymphocyte ratio; SII, New systemic immune inflammatory index; L1, Distal 1/3 ileum ± limited ileocecal disease; L2, Colon; L3, Returning colon; B1, Non narrow and non penetrating diseases; B2, Narrowing disease; B3, Penetrating diseases; B2B3, Narrow and penetrating diseases; PNR, Primary loss of response; LOR, Lost response.

### 3.4 Dynamic changes in body composition before and after treatment

All patients received IFX and EEN/PEN treatment. Among them, 44 patients required re-evaluation with CTE for disease assessment, with an average interval of 203.5 days between evaluations. After treatment, there were no significant changes in height or weight, however, BMI, L_3_SMI, and the L_3_SMI Z-score increased significantly (*P* < 0.05). Additionally, the proportion of patients with sarcopenia decreased significantly, from 47.7% to 25% (*P* = 0.027) (see Table 5).

**Table 5:** Dynamic changes in body composition before and after treatment.

**Variable**	**Before treatment (n = 44)**	**After treatment (n = 44)**	**P value**
Height [M(IQR), meter]	149.5 (135.2–157)	151.5 (136.3–160.7)	0.337
Weight [M(IQR), kg]	34.75 (26.1–42.3)	37.85 (29.4–48.75)	0.104
BMI [M(IQR), kg/m^2^]	15.78 (14.1–17.76)	17.04 (14.87–19.56)	0.043
L_3_SMI [M(IQR), cm^2^/m^2^]	24.66 (21.26–32.25)	30.04 (24.94–36.79)	0.038
L3SMI Z-score [M(IQR)]	−1.94 (−2.7 to −0.93)	−1.43 (−1.4 to −0.48)	0.048
Sarcopenia, n (%)	21 (47.7%)	11 (25%)	0.027

## 4 Discussion

This study demonstrates that sarcopenia is commonly observed in children with CD in China, with a significantly higher incidence compared to control children. Children with CD and sarcopenia show poorer nutritional status and increased inflammation-related markers, which are associated with a higher rate of non-response to the biologic therapy IFX. Treatment with IFX can improve the nutritional status of these children and reduce the incidence of sarcopenia.

Sarcopenia is more common among the elderly (36); however, recent studies have shown that secondary sarcopenia also frequently occurs in adults and children with various diseases, including endocrine disorders, tumors, liver diseases, and inflammatory bowel disease, etc. (7–28). The prevalence of sarcopenia in adults with IBD ranges from approximately 2% to 30% (37–39), while in pediatric CD, it ranges from 23.5% to 81% (18, 23). Consistent with other pediatric studies, our research also found sarcopenia in children with CD, with the incidence of 33.8%. Variations in incidence rates may be attributed to differences in study populations and the criteria used to assess skeletal muscle index. Additionally, we observed sarcopenia in healthy children in China, with an incidence rate of 10.2%, which may be related to reduced physical activity due to prolonged sedentary behavior and academic stress. Similar to Gülşen's findings (5), we also found sarcopenia during the remission phase of CD. Therefore, it is important to pay attention to the nutritional status of children with CD even during remission.

In addition to malnutrition, sarcopenia can also occur in patients with a normal BMI or obesity. A meta-analysis of 50 studies involving 86,285 individuals aged 60 years and older found that sarcopenic obesity has a prevalence of 11% among the Asian–Oceanic population (40). Naruse et al. reported that 9.6% (10 out 104) of CD patients had sarcopenic obesity (41). Although the exact cause remains unknown, but factors such as steroid therapy and lack of physical activity can lead to significant changes in body weight and contribute to sarcopenic obesity in IBD patients (42). In our study, we also identified seven patients with normal BMI or obesity who had sarcopenia, which suggests patients with a normal BMI or obesity can also develop sarcopenia, indicating that assessing the nutritional status of CD patients should not rely solely on BMI.

Currently, there is limited research on the risk factors associated with sarcopenia (43). A meta-analysis shows that gender, low BMI, age, and low albumin levels significantly affect the occurrence of sarcopenia in adult patients with CD (44). In this study, univariate analysis revealed that BMI, NLR, PLR, NMLR, and SII were linked to sarcopenia; however, binary logistic regression analysis identified low BMI as the sole risk factor. Low BMI is associated with malnutrition, which in turn increases the incidence of sarcopenia. No significant associations were found between gender, age, low albumin levels, and sarcopenia.

The diagnosis of adult sarcopenia is based on decline in skeletal muscle mass combined with low muscle strength and/or reduced physical performance (45). However, there is currently no unified definition or diagnostic criteria for sarcopenia in children (30, 31). Most studies diagnose pediatric sarcopenia using skeletal muscle mass indicators, such as the total psoas muscle area measured by CT or MRI (46). The total skeletal muscle area assessed by CT or MRI at the L3-L4 lumbar spine levels is considered the gold standard for measuring skeletal muscle mass (45). Although CT and MRI are expensive and lack portability, they are the fundamental methods for diagnosing CD and do not require additional examinations, making them more suitable than other methods for evaluating the skeletal muscle mass in CD patients. Compared to MRE, CTE is more appropriate for diagnosing and assessing pediatric CD in developing countries; therefore, CTE was used to evaluate skeletal muscle mass in this study. Reference standards for skeletal muscle mass vary by race and region. Based on CT scans, some researchers have reported pediatric reference values for the total lumbar muscle area in South Korea (47), Europe,and America (48), as well as reference values for the abdominal skeletal muscle compartments in Asian (49); Using DXA, Mi et al. reported pediatric reference values for skeletal muscle mass in China (50). Our study primarily relies on CTE to assess skeletal muscle area. Due to the lack of CT-based reference values for abdominal skeletal muscle area in Chinese children, Kudo et al.'s (49) standards were used in this study. Although some data on healthy children were collected in our study, the sample size was insufficient to develop a standard reference curve. Therefore, further multicenter studies are necessary to increase the sample size and improve CT-based reference values for abdominal skeletal muscle area in Chinese children. Muscle strength measurement in children primarily focuses on grip strength, with only one study addressing muscle strength in children (51). Given the challenges in assessing strength and function in young children, most pediatric studies do not include this information. As our study is retrospective, no muscle strength assessments were conducted. Future prospective studies will incorporate muscle strength measurements.

Sarcopenia may be associated with poor prognosis in CD, but there is still controversy. A meta-analysis indicated that sarcopenia is associated with an increased risk of hospitalization and abscess formation in patients with CD. However, it does not appear to significantly influence the need for surgery, loss of biological response, requirement for biological therapy, or the occurrence of surgical site leaks (52). Conversely, some studies has identified sarcopenia as a risk factor for developing LOR in CD patients treated with IFX (53–55). Research on pediatric populations is limited; for instance, Calia et al. found no significant association between sarcopenia and CD outcomes (19), whereas Atalan et al. (22) reported that patients with a psoas index in the lowest quartile had a significantly higher risk of requiring biologic therapy and experiencing disease exacerbation. Our study observed that CD patients with sarcopenia had a higher incidence of LOR and poorer outcomes when treated with IFX, although sarcopenia was not an independent risk factor for LOR. Larger studies are needed to better understand the impact of sarcopenia on CD outcomes in children.

The treatment of sarcopenia involves resistance training, nutritional support, and etiological treatment. In CD, treatment includes enteral nutrition support and immunosuppressive or biologic therapies, which improve both the nutritional status and inflammatory response of children, thereby reducing the incidence of sarcopenia. Subramaniam et al. (28) found that IFX can reverse inflammatory sarcopenia in patients with Crohn's disease. This study also supports that IFX combined with nutritional support can improve the nutritional status of children with CD and reverse sarcopenia. Research has shown that exercise can increase muscle mass in patients with IBD (56); however, due to low self-esteem, body image, and active IBD symptoms, many CD patients have limited physical activity (57). Consequently, some children with CD are unable to fully overcome sarcopenia through medication and nutritional support treatment alone. This study further demonstrated that after one year of treatment, 25% of patients still exhibited sarcopenia, highlighting the importance of incorporating appropriate exercise as part of the treatment plan for children with CD.

As one of the largest PIBD medical centers in China, this study is the first to use CTE to assess the prevalence of sarcopenia in Chinese children with CD and its impact on treatment outcomes Additionally, it longitudinally examines the reciprocal relationship between CD treatment and sarcopenia. However, this study has several limitations: (1) the absence reference standard range for total skeletal muscle area measured by CT/MRI at the L3-L4 lumbar spine levels in Chinese children;(2) its retrospective design, which lacks assessments of muscle strength, muscle function, and physical activity; and (3) a small sample size of CD patients meeting the inclusion criteria, potentially introducing selection bias. Further studies with larger cohorts are needed.

## 5 Conclusion

Sarcopenia is commonly observed in Chinese children with CD and can influence the effectiveness of biologic therapies. CTE can be assess CD-related sarcopenia in pediatric patients. Sarcopenia may be added to disease activity scores. IFX can improve the nutritional status of children with CD and reverse sarcopenia. However, due to limited awareness of this condition among pediatricians, further research is needed to better understand the relationship between sarcopenia and CD. Enhancing pediatricians' knowledge will facilitate early detection and intervention, ultimately improving the clinical outcomes for children with CD.

## Data Availability

The data presented in this study are available on request from the corresponding authors. The data are not publicly available due to privacy reasons. Requests to access these datasets should be directed to Jie Wu, wujie_022024@163.com.
